# Research Progress of Drug Delivery Systems Targeting the Kidneys

**DOI:** 10.3390/ph17050625

**Published:** 2024-05-13

**Authors:** Li-Feng Huang, Qiao-Ru Ye, Xiao-Cui Chen, Xiao-Rong Huang, Qiao-Fei Zhang, Chun-Yu Wu, Hua-Feng Liu, Chen Yang

**Affiliations:** Guangdong Provincial Key Laboratory of Autophagy and Major Chronic Non-Communicable Diseases, Key Laboratory of Prevention and Management of Chronic Kidney Disease of Zhanjiang City, Institute of Nephrology, Affiliated Hospital of Guangdong Medical University, Zhanjiang 524001, China; huanglifeng-1@outlook.com (L.-F.H.); Yqr134@outlook.com (Q.-R.Y.); chenxiaocui2012@outlook.com (X.-C.C.); HXR19930617@outlook.com (X.-R.H.); zhangqf@gdmu.edu.cn (Q.-F.Z.); wccyy666@hotmail.com (C.-Y.W.)

**Keywords:** chronic kidney disease, acute kidney injury, glomerulus, tubule, nanotechnology

## Abstract

Chronic kidney disease (CKD) affects more than 10% of the global population, and its incidence is increasing, partially due to an increase in the prevalence of disease risk factors. Acute kidney injury (AKI) is an independent risk factor for CKD and end-stage renal disease (ESRD). The pathogenic mechanisms of CKD provide several potential targets for its treatment. However, due to off-target effects, conventional drugs for CKD typically require high doses to achieve adequate therapeutic effects, leading to long-term organ toxicity. Therefore, ideal treatments that completely cure the different types of kidney disease are rarely available. Several approaches for the drug targeting of the kidneys have been explored in drug delivery system research. Nanotechnology-based drug delivery systems have multiple merits, including good biocompatibility, suitable degradability, the ability to target lesion sites, and fewer non-specific systemic effects. In this review, the development, potential, and limitations of low-molecular-weight protein–lysozymes, polymer nanomaterials, and lipid-based nanocarriers as drug delivery platforms for treating AKI and CKD are summarized.

## 1. Introduction

Chronic kidney disease (CKD) affects more than 10% of the global population, and its incidence is increasing, partially due to increases in the prevalence of disease risk factors, such as diabetes, hypertension, obesity, and old age [1]. Acute kidney injury (AKI) is a syndrome with multiple etiologies [2] that affects approximately 1.33 million patients per year, leading to a significant impact on hospitalization and mortality rates, especially in severely ill patients [3]. AKI is a risk factor for CKD and accelerates its progression to ESRD [4].

Immune dysfunction and renal inflammation are the major pathological features of CKD that aggravate renal function loss. Hormones and immunosuppressants are common forms of medication used to treat CKD that suppress renal inflammation. However, the long-term use of high concentrations of these drugs is associated with adverse effects and inefficiency [5,6,7]. The known pathogenic mechanisms of CKD provide several potential therapeutic targets; however, some targets are cell specific. Transforming growth factor-β (TGF-β) plays a key role in triggering renal fibrosis during the development of CKD and is positively correlated with the severity of the disease [8]. Although anti-TGF-β treatment inhibits the pro-fibrotic effects of renal resident cells, such as renal tubular epithelial cells and fibroblasts, the loss of TGF-β may lead to the hyperactivation of immune cells and increase renal inflammation [9]. Similarly, renin–angiotensin system blockade delays the progression of proteinuria and hypertensive nephropathy [1,10]. However, the stimulation of AT1 in macrophages or T cells attenuates renal fibrosis [11,12]. In summary, the off-target effects of existing therapeutics limit their use for the treatment of different kidney diseases. Therefore, cell-specific drug delivery systems may provide suitable therapeutic approaches for AKI and CKD.

### Overview of Targeted Drug Delivery Systems

Targeted drug delivery systems (TDDSs) are drugs encapsulated in a carrier that can be selectively concentrated and localized into target organs, tissues, cells, or intracellular structures via topical administration or systemic blood drug circulation. For drugs to be considered as TDDSs, they require five key characteristics: positioning (releasing the drug at the specified location), controlled release [13], non-toxicity, biocompatibility, and biodegradability [14]. The primary purpose of targeted drug administration is to improve drug efficacy and reduce the rate of adverse effects [15]. Improvements in dosing strategies also improve safety, effectiveness, reliability, and patient compliance; therefore, TDDSs are increasingly valued by the global medical community.

**Figure 1:**
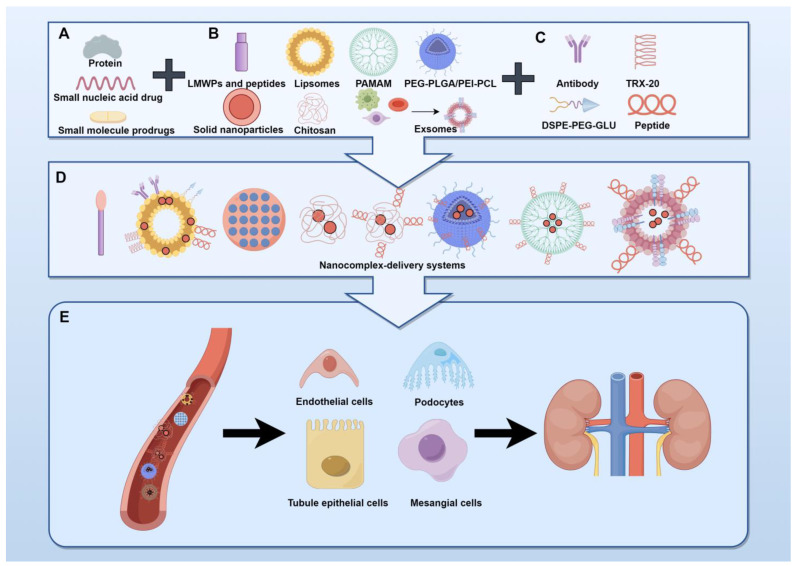
Targeted drug delivery systems: (**A**) deliverable medications; (**B**) different types of carriers; (**C**) different target ligands; (**D**) different strategies of drug delivery systems; and (**E**) targeting kidney. Abbreviations: DSPE-PEG-GLU: phospholipid–polyethylene glycol–glucose; LMWPs: low-molecular-weight proteins; PLGA: poly lactic-co-glycolic acid; PAMAMs: polyamidoamine dendrimers; and TRX-20: 3,5-dipentadecyloxybenzamidine hydrochloride. The figure was drawn by Figdraw (www.figdraw.com), accessed on 25 April 2024.

Based on the various targeting mechanisms available, targeted drug delivery can be categorized as passive or active or as physical chemistry targeting [13]. Passive targeted drug delivery utilizes the physiological characteristics of specific tissues and organs to naturally create distribution differences in the body, thereby achieving a targeting effect. Active targeted drug delivery refers to drugs or carriers endowed with abilities to combine with special targets. Active TDDSs include three main components: drugs, carriers, and targeted ligands. The encapsulated drugs include small-molecule proteins, small-molecule nucleic acids, or small-molecule precursor drugs (Figure 1A). With the advancement of nanotechnology, an increasing number of beneficial nanomaterials have been developed. Nanomaterials have been utilized as carriers for drug delivery in various studies, including chitosan [16,17], poly lactic-co-glycolic acid (PLGA) [18,19], lipid nanocarriers [20], and polyamidoamine dendrimers (PAMAMs) [21]. Additionally, small molecule peptides, such as low-molecular-weight proteins (LMWPs) [22] and elastin-like polypeptides (ELPs) [23], can also serve as effective carriers. Furthermore, the use of cell-derived extracellular vesicles as drug delivery vehicles is a current area of research interest [24] (Figure 1B). The use of drugs encapsulated by carriers can enhance their effects by improving their absorption and lowering their dosages, resulting in fewer adverse effects than traditional medicine [25]. Similarly, the encapsulation of drugs by these carriers has been shown in other studies to enhance their effects by improving absorption and reducing dosages, thereby minimizing the rate of adverse effects compared to traditional medicine [25,26,27]. Targeted ligands are specific functional groups and biologically active molecules [28] including peptides, antibodies, TRX-20, and DSPE-PEG-GLU (Figure 1C). Chemical bonds connect the carrier that encapsulates the drug, forming a targeting complex to direct its action to the kidney (Figure 1D,E). Physical chemistry targeting involves the application of various physical and chemical methods to facilitate the targeted delivery of therapeutic agents for specific drug effects at a particular site [29].

## 2. Kidney-TDDS

### 2.1. LMWPs and Small-Molecule Peptide TDDSs

#### 2.1.1. LMWP–Lysozyme

**Figure 2:**
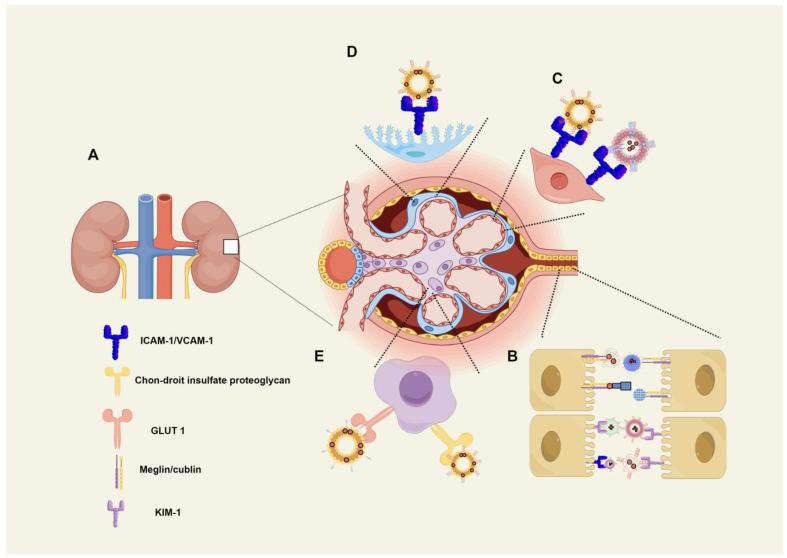
Drugs absorbed via different receptors: (**A**) the structure of the glomeruli in the kidneys is shown; (**B**) nanocomplex absorbed via VCAM-1, ICAM-1, megalin/cubilin, and the KIM-1 receptor in renal tubular epithelial cells; (**C**) nanocomplex absorbed via VCAM-1 receptors in endothelial cells; (**D**) nanocomplex absorbed via VCAM-1 receptors in podocytes.; and (**E**) nanocomplexes absorbed by GLUT1 and chondroitin sulfate proteoglycan receptors in mesangial cells. Abbreviations: ICAM-1: intercellular adhesion molecule-1; VCAM-1: vascular cell adhesion molecule-1. The figure was drawn by Figdraw (www.figdraw.com), accessed on 25 April 2024.

The glomerular filtration barrier includes charge and molecular barriers that prevent large molecules from passing from the blood into the urine (Figure 2A). LMWPs with molecular weights of <30 kDa can cross the glomerular filtration barrier freely and are absorbed by the proximal tubules via megalin/cubilin-mediated endocytosis (Figure 2B). Lysozymes (LZM) weighing 14.3 kDa are considered non-immunogenic [30] and are ideal carriers for the delivery of drugs to the proximal tubules [31].

Imatinib, an anticancer drug, can also be used to treat renal interstitial fibrosis, as it inhibits platelet-derived growth factor receptors (PDGFRs) and Abelson tyrosine kinases (c-Abl) [32]. Dolman et al. prepared a kidney-targeted LZM–imatinib complex using a platinum-based linker that accumulates in renal proximal tubular cells 30-fold more accurately than imatinib in equal doses without any cardiotoxicity [33]. Baicalin (BAI) is a Chinese herbal medicine that reduces insulin resistance and protects the kidneys. In their recent study, Zheng et al. synthesized a BAI-LZM conjugate that mitigated diabetic nephropathy (DN) better than BAI alone in a rat model through the inhibition of inflammation by targeting NF-kB signaling [34]. In a similar manner, Pan et al. covalently connected methylprednisolone (MP) with LZMs via an ester bond, increasing the targeting efficiency of active MP by approximately 14 times compared to that of free MP. Moreover, the use of tissue imaging in their study showed that the conjugate quickly reaches the kidney and continues acting in the kidney for up to 12 h [35]. Not only do LZMs improve drug utilization, but also they reduce the rate of adverse effects caused by the drug in question due to their excellent targeting ability. Nevertheless, further optimization is necessary before the commercial production and purification of LZMs [36].

#### 2.1.2. Peptides

Peptides are highly selective, effective, and relatively safe [37]. Engel et al. constructed a complex that modified ELP–vascular endothelial growth factor (VEGF) with the inactive peptide KTP (CLPVASC) to target renal tubules. Compared with ELP-VEGF, KTP-ELP-VEGF was found to mitigate renal vascular stenosis and improve filtration functions in a swine chronic renovascular disease (RVD) model by inhibiting macrophage infiltration and reducing fibrosis [38]. Basic fibroblast growth factors (bFGFs) were found to reduce apoptosis and excessive endoplasmic reticulum stress in a renal ischemia–reperfusion (I/R) model [39]. However, they easily diffuse into the body, significantly reducing the drug concentration in the kidneys and leading to adverse effects. In their recent study, Song et al. prepared a targeted injured kidney (KIT, CNWMINKEC)-bFGF compound that utilized KIT to bind to kidney injury molecule-1 (KIM-1), a transmembrane glycoprotein that is highly expressed in damaged tubules during I/R-induced AKI. KIT-bFGF significantly reduced tubular apoptosis and protected against renal I/R through the ERK1/2 and Akt signaling pathways [40]. Compared to biologics and small molecules, peptides have the advantages of permeability, high selectivity, and ease of internalization by cells [41]. However, one disadvantage of peptides is that they are easily cleared from the body due to their low molecular weight (<5000 Da).

### 2.2. Lipid-Based Nanocarriers

A number of different lipid-based systems have been reported in the literature, including liposomes, micelles, emulsions, and solid lipid nanoparticles (SLNSs) [42]. They are reported to have good biocompatibility, biodegradability, low toxicity, and immunogenicity and are reported to be easy to modify through the use of various ligands and functional molecules.

Liposomes, a commonly used nanocarrier system, are tiny, hollow spherical structures composed of one or two phospholipid molecules with a bilayer membrane structure. Due to their unique structure, liposomes are lipophilic and hydrophobic, making it simple for liposomes to encapsulate water-soluble and lipid-soluble drugs. Mesangial cells are a type of glomerular intrinsic cells that are involved in a variety of biological responses associated with glomerular disease. Liposomes modified with TRX-20 were found to demonstrate specific targeting toward mesangial cells by binding to chondroitin sulfate proteoglycan [43] (Figure 2E). Yuan ZX et al. used TRX-20 to modify the liposomes that encapsulated the drug triptolide (TP) to form a TRX-TP-LP complex [44]. To prolong the circulation of the complex in the blood, PEG was used to further modify the surface of TRX-TP-LP to form PEG-TRX-TP-LP to target mesangial cells in glomeruli. Compared to the equivalent dose of free TP, PEG-TRX-TP-LP attenuated LPS-induced inflammatory responses in vitro. In addition, in vivo, PEG-TRX-TP-LP effectively alleviated the symptoms of membranous nephropathy (MN) in a rat model. Astaxanthin (AST) is a natural, non-toxic xanthophyll carotenoid with antioxidant capacity. Its low stability and solubility severely limit its application. Oxidative stress is one of the main pathological mechanisms caused by diabetic nephropathy. In their recent study, Chen Z et al. used a glucose-PEG600-DSPE ligand to modify liposomes coated with AST to form an AST-GLU-LIP complex, which specifically transports AST by interacting with glucose transporter 1 (GLUT1), which is highly expressed in glomerular mesangial cells in DN [45] (Figure 2E). The findings of their study indicated that AST-GLU-LIP enhanced the bioavailability and antioxidant capacity of AST. Microvascular endothelial cells play a crucial role in the pathogenesis of sepsis-induced inflammation-mediated organ damage, including kidney injury. Although glucocorticoids have anti-inflammatory effects, they do not have the therapeutic potential to alleviate endothelial cell activation during sepsis due to inadequate drug delivery. In their recent study, Li et al. combined the cationic lipid SAINT-C18 (1-methyl-4-(cis-9-dioleyl) methyl-pyridinium chloride), termed SAINT-O-Somes, with dexamethasone. The SAINT-O-Somes were modified with anti-vascular cell adhesion molecule-1 (anti-VCAM-1) antibodies. Dexamethasone-loaded anti-VCAM-1 SAINT-O-Somes specifically targeted VCAM-1-expressed endothelial cells and effectively reduced inflammation in the kidneys during sepsis [46] (Figure 2C). Cationic liposomes may pose a significant disadvantage when administered intravenously, as this form of administration can lead to high levels of toxicity and a lack of specific targeting [28].

Solid lipid nanocarriers are generally prepared from degradable and biocompatible components. They are novel drug carriers with long release time, low toxicity, and better cellular absorption ability than traditional colloidal carriers. Antioxidants such as phenolic compounds have several benefits for the human body [47] and can be used to treat type 2 diabetes mellitus and DN via the inhibition of reactive oxygen species generation [48]. Myristicin is a polyphenolic compound and flavanol glycoside derived from plants that can lower blood sugar and exert anti-apoptotic and anti-inflammatory activities and antioxidant functions [49]. However, it has low bioavailability due to its poor water solubility, thus limiting its application [50]. Ahangarpour et al. formed solid lipid nanoparticles (SLNs) of myricitrin via cold homogenization. Compared with free myristicin, SLNs with myristicin have a better therapeutic effect on DN by inhibiting oxidative stress and increasing antioxidant enzyme production [51]. Celastrol (CLT) is an active ingredient in Tripterygium Wilford that is effective for the treatment of DN but is limited by its toxicity [52]. Endothelial injury and podocyte damage are the major causes of glomerulosclerosis. Wu et al. synthesized CLT–phospholipid nanoparticles (PLNs) and covered the CLT-PLN surface with a VCAM-1-targeting peptide (sequence: VHPKQHRGGSKGC) that specifically released CLT to VCAM-1-positive glomerular endothelial cells and podocytes [53] (Figure 2C,D). CLT-PLN effectively reduced podocyte and endothelial damage and the glomerular inflammatory response, leading to delayed CKD progression with almost no CLT toxicity. These nanoparticles provide an effective delivery strategy to the glomerulus. In addition, lipid-based nanocarriers are excellent carriers for drug delivery due to their biocompatibility, minimal toxicity, degradability, and ease of modification. Most importantly, large-scale production is the greatest advantage of the use of lipid-based nanocarriers relative to other polymer materials [54,55]. However, the synthesis of lipid-based carriers, as well as their size and charge, is important for the kidneys, limiting their ability to achieve targeted drug delivery [56,57]. Maintaining a stable carrier size and selecting an appropriate charge are key factors in targeted drug delivery. Solid lipid nanocarriers still have some shortcomings that need to be addressed, such as the large amount of water present in these nanocarriers, their limited drug-loading capacity, and the possible formation of crystals in solid lipids during storage [58].

### 2.3. Polymeric Nanoparticle TDDSs

Polymeric materials have attracted increasing attention in medical biology research due to their biodegradability, controlled release, extended cycle time, low toxicity, and ability to carry drugs [18].

#### 2.3.1. Chitosan

Chitosan is the primary derivative of chitin [59]. Chitin is converted into chitosan via enzymatic preparations or chemical processes [60]. Chemical methods are more suitable for large-scale production due to their low cost [60]. Chitosan carriers include polysaccharides and have a mechanism similar to that of ELPs [61]. Szeto-Schiller-31 (SS31) is a mitochondria-targeted peptide that reduces mitochondrial damage by scavenging free oxygen radicals [62]. Liu et al. synthesized SC-TK-SS31 by combining SS31 with l-serine-modified chitosan (SC) using a reactive oxygen species-sensitive thioketal (TK) linker. Through the modification of L-serine, SC-TK-SS31 accumulates in tubular epithelial cells via the specific interaction of L-serine with KIM-1 (Figure 2B), enhancing the therapeutic effect of SS-31 [63]. Wang et al. constructed a stepwise-targeted chitosan oligosaccharide, triphenyl phosphine low-molecular-weight chitosan–curcumin (TPP-LMWC-CUR, TLC), which was used to treat AKI induced by sepsis by clearing excessive reactive oxygen species in tubular epithelial cells. TLC is quickly distributed in the kidneys and specifically internalized by the renal tubules through interactions between the receptor megalin and LMWCs. Due to the high buffering capacity of LMWCs and the positive delocalization charge of TPPs, intracellular TLCs can deliver CURs to the mitochondria [64]. However, chitosan production is expensive. In addition, it is easily affected by several factors, and the efficiency of dissolution is poor, which may account for the low yield. Therefore, the problem of standardized production requires further research efforts for a solution [65].

#### 2.3.2. Polyamidoamine Dendrimers

In the late 1990s, polyamidoamine dendrimers (PAMAMs) were extensively studied as drug carriers due to their ease of preparation and commercial availability [66]. PAMAMs decorated with neutral or negatively charged groups have been reported to reduce the toxicity caused by strong electrostatic interactions between PAMAMs and cell membranes [67]. PAMAMs increase drug solubility, stability, and bioavailability, facilitate synthesis methods, and are promising dendrimers in the field of drug delivery [68]. Serine-modified PAMAMs are a promising delivery vehicle that target renal tubules (Figure 2B).

Captopril (CAP) is an angiotensin-converting enzyme (ACE) inhibitor associated with rare cases of acute liver injury. Matsuura et al. combined captopril with a disulfide bond in l-serine-modified PAMAMs. In addition, compared to the non-targeted group, up to 82% of the dose of serine-PAMAM-CAP accumulated in the kidneys, and the duration of ACE inhibition was longer than that after the injection of CAP alone [69].

Nitric oxide (NO) contributes to the pathogenesis of renal I/R injury (Schneider et al., 2003, https://www.kidney-international.org/article/S0085-2538(15)49309-3/fulltext). NO donors are physiologically active drugs that release NO into the body. However, the efficient prevention of renal I/R injury requires large amounts of NO donors, and most existing NO donors are rarely distributed in the kidney [70,71]. Katsumi et al. developed S-nitrosyl-L-serine-modified PAMAMs (SNO-Ser-PAMAMs), in which multiple S-nitrosothiols (NO donors) are covalently bound to L-serine-modified dendrimers as kidney-targeted NO donors [72]. In a murine model of renal ischemia–reperfusion (I/R) injury, the intravenous administration of SNO-Ser-PAMAMs effectively attenuated histological damage in the kidneys and reduced blood creatinine levels [72]. Matsuura et al. and Katsumi et al. reported that wrapping CAP and NO donors with dendritic macromolecules with a specific binding modification of serine to KIM-1 localized the drug to the kidneys and increased the retention of the drug in the kidneys without any toxicity. The flexibility and biocompatibility as well as the biodegradability of PAMAMs make them an attractive option for drug delivery [73]. However, there are specific concerns that require further attention. Firstly, unmodified PAMAM materials with positively charged surfaces are generally considered toxic [74]. Secondly, smaller PAMAMs can be easily eliminated by the kidneys; in contrast, larger ones tend to be recognized by the reticuloendothelial system (RES) [75]. To mitigate these effects, surface modifications have been employed to improve both the toxicity profile of the vector and its clearance rate [68]. The toxicity of dendrimers can be reduced by acetylating and PEGylating peripheral positively charged groups to modify them. This approach helps to mitigate the toxic effects of dendrimers, making them a more viable option for various applications [76].

#### 2.3.3. PEG-PLGA/PEI-PCL

PLGA has been approved for use as a drug delivery system in humans by the US Food and Drug Administration and the European Medicine Agency [77]. PLGA-based nanoparticles are easily captured and removed by the reticuloendothelial system following intravenous injection [78]. Polyethylene glycol (PEG) is non-toxic and soluble in water and organic solvents. It has no immunogenicity and is ultimately excreted through the kidneys, making it a suitable drug carrier. Materials such as PLGA-containing PEG chain segments have been designed to regulate their degradation and the release behavior of drugs by controlling their hydrophilic/hydrophobic properties [79]. The use of PEG to modify PLGA increases the overall cycle time and bioavailability.

Asiatic acid (AA) is a triterpenoid compound primarily found in the medicinal plant Centella asiatica. It exhibits a range of biological activities, including anti-inflammatory, anticancer, hepatoprotective, renoprotective, and anti-fibrotic effects [22,80,81]. However, the limited water solubility of AA restricts its application. He et al. used the peptide KTP (sequence: CLPVASC)-modified PLGA-PEG as a drug carrier and wrapped insoluble AA in a vehicle, which targeted renal tubular epithelial cells via megalin-mediated endocytosis (Figure 2B), resulting in protection against renal fibrosis [82]. Toll-like receptors (TLRs) are a type of pattern recognition receptors that play a crucial role in regulating both innate and adaptive immunity [83,84]. The results of a previous study showed that mice with selective knockout of TLR9 in the renal proximal tubules were protected against I/R-induced AKI [85]. Therefore, a selective TLR9 antagonist (ODN2088) could be considered a promising drug for the treatment of AKI. However, TLR9 is expressed in several cells and organs [86] and may be accompanied by diverse effects. A recent study reported that mesoscale nanoparticles based on polymers exhibit a seven-fold greater localization in the proximal tubules of the kidney compared to other organs. This finding suggests a potential targeting advantage for these in renal drug delivery applications [87]. Han et al. packaged the selective TLR9 antagonist ODN2088 into medium-sized PLGA-PEG nanoparticles. Through the endocytosis of tubules, they release the TLR9 antagonist ODN2088 and suppress TLR9 to relieve tubular necrosis and the renal production of pro-inflammatory cytokines caused by I/R reperfusion [88].

Polycaprolactone–polyethyleneimine (PCL-PEI) is a carrier material that can be used for the co-administration of nucleic acid and small-molecule chemical drugs. This polymer can form cationic micelles via self-assembly and load negatively charged drugs, such as nucleic acids, based on the action of the charge. Rhein is a monomeric compound isolated from rhubarb and is a traditional Chinese medicine. It has been found to possess antioxidant, anti-apoptotic, and anti-inflammatory properties that can effectively protect the kidneys; however, its low solubility and poor bioavailability in vivo limit its clinical application [89]. Wang et al. first wrapped Rhein in PCL-PEI to form PRR nanoparticles. The surface of PRR nanoparticles is covered with a layer of liposomes modified by KTP through the action of electrostatic attraction in the PRR to form kidney-targeted lipid nanoparticles (KLPPRs). With the modification of KTP, KLPPR accumulates in the kidneys to a greater level than in other major organs (Figure 2B). In addition, KLPPR is not as easily cleared in the urine as LPPR. Therefore, the use of KLPPR is an effective method for enhancing the distribution and retention of Rhein in the kidneys, as well as increasing its solubility and bioavailability [90].

Although the surface modification of PLGA and PCL can improve the bioavailability of drugs and reduce the rate of side effects caused by said drugs, there are still some challenges that must be addressed. One is developing a novel method to achieve the large-scale production of high-quality drugs [91]. Additionally, limited drug-loading capacity may impose restrictions on their application [92].

### 2.4. Exosome TDDS

Exosomes are extracellular vesicles secreted by all cell types that are capable of carrying small molecules such as nucleic acids and small protein molecules [93]. Exosome-based treatment utilizes the genetic material carried by the exosome to repair the lesion site. Alternatively, exosomes can serve as natural drug delivery vehicles, transporting specific drugs to the lesion site through modifications [94].

Mesenchymal stromal cells (MSCs) possess the ability to self-renew and facilitate repair [95]. It has been reported that exosomes derived from MSCs (referred to as MSC-exos) have demonstrated an ability to mitigate cisplatin-induced acute kidney injury (AKI) by inhibiting oxidative stress and cell apoptosis [96]. Cao et al. reported that MSC-exos are home to damaged renal tubules expressing VCAM-1 and intercellular adhesion molecule 1 (ICAM-1) during renal I/R injury via integrins [97] (Figure 2B). MiR-125b-5p/p53 has been reported to be the main molecule present in stem cell-derived exosomes that reduces AKI and promotes kidney repair. MSC-exos have a favorable safety profile. In addition, MSC-exos can be removed by the immune system once they have exerted their function; considering this, the potentially tumorigenic effects of MSCs can be considered negligible. However, the possibility of variance between different individuals leads to unstable efficacy; as such, personalized treatment is required.

Apart from MSC-exos, engineered exosomes are a class of modified exosomes that have enhanced drug-loading efficiency, targeting, and resistance to the clearance of natural exosomes after bioengineering technology. Tang et al. incubated dexamethasone with RAW264.7 macrophages to induce the secretion of dexamethasone-containing exosomes and used integrin on the extracellular vesicle surface to specifically bind to ICAM-1 and VCAM-1 to achieve anti-inflammatory effects and reduce the rate of side effects caused by dexamethasone [98] (Figure 2B). In addition to dexamethasone, interleukin (IL)-10 was loaded onto macrophage-derived exosomes by specifically binding to ICAM-1. The extracellular vesicles delivering IL-10 enhance IL-10 stability and target the damaged kidney, resulting in significant renal protection (Figure 2B). These results emphasize the significant feasibility of targeting ICAM and drug vectors [94,99]. Erythrocyte-derived exosomes modified with peptides (sequence: LTHVVWL) that specifically bind to KIM-1 combined with p65 and Snail-1 siRNA loaded into exosomes can be used to treat AKI [100] (Figure 2B).

Compared to other targeted drug delivery systems, exosomes exhibit exceptional biocompatibility and minimal immunogenicity owing to their endogenous nature [101,102]. However, their utilization still encounters certain challenges. Despite the availability of various sources for deriving exosomes, there is a lack of standardized methods for their separation and extraction to ensure optimal purity [101,103,104,105]. Moreover, enhancing the loading efficiency of exosomes remains a formidable task. Meeting clinical translational needs necessitates addressing the arduous problem of producing high-quality and large-scale vectors for disease treatment [103].

## 3. Challenge and Perspective

Good biocompatibility, biodegradability, and low toxicity are essential features of a highly effective targeted drug delivery vector. In addition, drugs with poor solubility and adverse effects can be administered through carrier encapsulation. However, targeted drug delivery has limitations. Long-term drug use may lead to adverse events related to the stability and purity of the carrier. There is also difficulty in maintaining stable targeting efficiency during repeated dosing.

The specificity, stability, and efficacy of drug delivery require further research regarding targeted delivery strategies. Phage display technology and single-cell sequencing may be useful tools in efforts to discover target molecules or cell-specific ligands. Intelligent response targeting strategies can be designed according to changes in the microenvironment of kidney disease. Changes in targeting strategies and improvements in the durability, stability, and specificity of the vector also require further research.

## 4. Conclusions

The kidney, as a major organ responsible for the excretion of metabolites, receives about 25% of the total cardiac blood transfusion. The selection of targeted delivery vectors with reduced nephrotoxicity to minimize potential harm is essential for the successful translation of biotechnology into clinical applications. Targeted therapy of the kidney has demonstrated its potential for the treatment of different kidney diseases (Table 1). It remains necessary to find suitable drug delivery vectors and continuously optimize renal targeting strategies in the future.

## Figures and Tables

**Table 1:** Summary of drug delivery systems targeting kidney-resident cells in different kidney diseases.

Target Cell	Carrier	Drug	Application	Target Mechanism	Reference
Tubular epithelial cells	LMWP–lysozyme	Imatinib	Renal fibrosis	Megalin/cubilin-mediated endocytosis	[33]
Tubular epithelial cells	LMWP–lysozyme	BAI	DN	Megalin/cubilin-mediated endocytosis	[34]
Tubular epithelial cells	LMWP–lysozyme	MP	Improvement in targeting efficiency	Megalin/cubilin-mediated endocytosis	[35]
Tubular epithelial cells	Peptide–ELP	VEGF	RVD	Megalin/cubilin-mediated endocytosis	[38]
Tubular epithelial cells	Peptide–KIT	bFGFs	I/R-AKI	KIT specifically combined with KIM-1	[40]
Tubular epithelial cells	Solid lipid nanoparticles	Myristicin	DN	Endocytosis of tubules	[51]
Tubular epithelial cells	Chitosan	SS31	I/R-AKI	L-serine special combined with KIM-1	[63]
Tubular epithelial cells	LMWC	Curcumin	AKI induced by sepsis	Megalin-mediated endocytosis	[64]
Tubular epithelial cells	PAMAM	CAP	Improved targeting efficiency	L-serine specifically combined with KIM-1	[69]
Tubular epithelial cells	PAMAM	NO donors	I/R-AKI	L-serine specifically combined with KIM-1	[72]
Tubular epithelial cells	PEG-PLGA	AA	Renal fibrosis	Megalin-mediated endocytosis	[82]
Tubular epithelial cells	PEG-PLGA	ODN2088	I/R-AKI	Endocytosis of tubules	[88]
Tubular epithelial cells	PEG-PCL	Rhein	DN	Megalin-mediated endocytosis	[90]
Tubular epithelial cells	Mesenchymal stromal-cells exosomes	MiR-125b-5p/p5	I/R-AKI	VCAM-1 and ICAM-1 bind with integrin	[97]
Tubular epithelial cells	Macrophage-derived exosomes	IL-10	I/R-AKI	VCAM-1 and ICAM-1 bind with integrin	[94,99]
Tubular epithelial cells	Red blood cell-derived exosomes	Sip65, siSnail-1	I/R-AKI	Peptides specifically combine with KIM-1	[100]
Endothelial cells	Phospholipid nanoparticles	CLT	DN	Peptides combine with VCAM-1	[53]
Endothelial cells	Macrophage-derived exosomes	Dexamethasone	LPS-induced nephropathy and ADR-induced nephropathy	VCAM-1 and ICAM-1 bind with integrin	[98]
Podocyte	SAINT-O-Somes	Dexamethasone	Sepsis-induced AKI	VCAM-1 antibody combines with VCAM-1	[46]
Podocyte	Phospholipid nanoparticles	CLT	DN	Peptides combine with VCAM-1	[53]
Mesangial cells	Liposomes	TP	MN	TRX20 specifically binds to chon-droit in sulfate proteoglycan	[44]
Mesangial cells	Liposomes	AST	DN	Glucose ligands bind with GLUT1	[45]

Abbreviation: AA: asiatic acid; AKI: acute kidney injury; AST: astaxanthin; BAI: baicalin; bFGFs: basic fibroblast growth factors; CAP: captopril; CLT: celastrol; DN: diabetic nephropathy; GLUT1: glucose transporter 1; ICAM-1: intercellular adhesion molecule-1; I/R: ischemia–reperfusion; KIM-1: kidney injury molecule-1; LMWC: low-molecular-weight chitosan; MN: membranous nephropathy; MP: methylprednisolone; PAMAMs: polyamidoamine dendrimers; PCL: polycaprolactone; PEI: polyethyleneimine; PLGA: polylactic-co-glycolic acid; RVD: renovascular disease; SS31: Szeto-Schiller-31; TP: triptolide; VCAM-1: vascular cell adhesion molecule-1; and VEGF: vascular endothelial growth factor.

## Abbreviations

AA: asiatic acid; ACE: angiotensin-converting enzyme; AKI: acute kidney injury (AKI); AST: astaxanthin; BAI: baicalin; bFGFs: basic fibroblast growth factors; c-Abl: Abelson tyrosine kinase; CAP: captopril; CKD: chronic kidney disease; CLT: celastrol; CUR: curcumin; DN: diabetic nephropathy; DSPE-PEG-GLU: phospholipid–polyethylene glycol–glucose; ELP: elastin-like polypeptides; ESRD: end-stage renal disease; GLUT1: glucose transporter 1; I/R: ischemia–reperfusion: ICAM-1: intercellular adhesion molecule-1; KIM-1: kidney injury molecule-1; KLPPRs: kidney-targeted lipid nanoparticles; LMWC: low-molecular-weight chitosan; LMWPs: low-molecular-weight proteins; LZM: lysozymes; MN: membranous nephropathy; MSCs: mesenchymal stromal cells; MP: methylprednisolone; NO: nitric oxide; PAMAM: polyamidoamine dendrimers; PCL-PEI: polycaprolactone–polyethyleneimine; PDGFR: platelet-derived growth factor receptors; PEG: polyethylene glycol; PLGA: poly lactic-co-glycolic acid; RES: reticuloendothelial system; RVD: chronic renovascular disease; SLNs: solid lipid nanoparticles; SS31: Szeto-Schiller-31; TDDs: targeted drug delivery systems; TGFβ: transforming growth factor-β; TLC: triphenyl phosphine low-molecular-weight chitosan–curcumin; TLRs: Toll-like receptors; TP: triptolide; TRX-20: 3,5-dipentadecyloxybenzamidine hydrochloride; VCAM-1: vascular cell adhesion molecule-1; and VEGF: vascular endothelial growth factor.
